# Erythropoietin response in critically ill mechanically ventilated patients: a prospective observational study

**DOI:** 10.1186/cc3480

**Published:** 2005-02-25

**Authors:** Alan J DeAngelo, David G Bell, Michael W Quinn, Deborah Ebert Long, Daniel R Ouellette

**Affiliations:** 1 Physician, Pulmonary and Critical Care Service, Dwight David Eisenhower Army Medical Center, Fort Gordon, Georgia, USA; 2 Fellow, Pulmonary and Critical Care Service, Brooke Army Medical Center, Fort Sam Houston, Texas, USA; 3 Physician, Pulmonary and Critical Care Service, David Grant Air Force Medical Center, Travis Air Force Base, California, USA; 4 Pulmonary and Critical Care Service, Brooke Army Medical Center, Fort Sam Houston, and Assistant Program Director PCCM fellowship, Brooke Army Medical Center, Fort Sam Houston, Texas, USA

## Abstract

**Introduction:**

Anemia is a common problem in critically ill patients. The etiology of anemia of critical illness is often determined to be multifactorial in the clinical setting, but the pathophysiology remains to be elucidated. Erythropoietin (EPO) is an endogenous glycoprotein hormone that serves as the primary stimulus for erythropoiesis. Recent evidence has demonstrated a blunted EPO response as a factor contributing to anemia of critical illness in specific subsets of patients. Critically ill patients requiring mechanical ventilation who exhibit anemia have not been the subject of previous studies. Our goal was to evaluate the erythropoietic response to anemia in the critically ill mechanically ventilated patient.

**Methods:**

A prospective observational study was undertaken in the medical intensive care unit of a tertiary care, military hospital. Twenty patients admitted to the medical intensive care unit requiring mechanical ventilation for at least 72 hours were enrolled as study patients. EPO levels and complete blood count were measured 72 hours after admission and initiation of mechanical ventilation. Admission clinical and demographic data were recorded, and patients were followed for the duration of mechanical ventilation. Twenty patients diagnosed with iron deficiency anemia in the outpatient setting were enrolled as a control population. Control patients had baseline complete blood count and iron panel recorded by primary care physicians. EPO levels were measured at the time of enrollment in conjunction with complete blood count.

**Results:**

The mean EPO level for the control population was 60.9 mU/ml. The mean EPO level in the mechanically ventilated patient group was 28.7 mU/ml, which was significantly less than in the control group (*P *= 0.035). The mean hemoglobin value was not significantly different between groups (10.6 g/dl in mechanically ventilated patients versus 10.2 g/dl in control patients; *P *> 0.05).

**Conclusion:**

Mechanically ventilated patients demonstrate a blunted EPO response to anemia. Further study of therapies directed at treating anemia of critical illness and evaluating its potential impact on mechanical ventilation outcomes and mortality is warranted.

## Introduction

Critically ill patients frequently develop anemia during their intensive care unit (ICU) course. Corwin and coworkers [1] reported that 95% of patients demonstrated abnormal hemoglobin concentration by the third ICU day. Anemia in the ICU patient has been reported to resemble anemia of chronic disease in its metabolic pattern [2]. The etiology of anemia of critical illness is multifactorial; it often results from a combination of primary losses, abnormal coagulation, nutritional deficiencies, depressed bone marrow production, and phlebotomy. Recent evidence has demonstrated a blunted erythropoietin (EPO) response to be a factor contributing to anemia of critical illness in specific subsets of patients, including those with sepsis, multiple trauma, and pediatric critical illness [3-5]. The EPO response in adult patients requiring mechanical ventilation for respiratory failure has not been studied as a primary end-point.

EPO is an endogenous glycoprotein hormone that serves as the primary stimulus for erythropoiesis. The kidney is the primary site of EPO production, but the liver also produces the hormone. EPO acts in the bone marrow, where it promotes terminal differentiation of progenitor cells into erythrocytes [6]. Diminished arterial oxygen content associated with anemia or hypoxia is the major stimulus for EPO production and usually produces an exponential increase [7-9].

Anemia of critical illness and blood management strategies in the ICU continue to be areas of active research. Two recent trials [10,11] demonstrated a reduction in the number of transfusions in critically ill patients treated with recombinant human EPO (rHuEPO). Mortality and adverse clinical events were not statistically different between groups in either study. Hebert and coworkers [12] investigated the effects of a restrictive (threshold 7 g/dl, goal 7–9 g/dl) versus a liberal (threshold 10 g/dl, goal 10–12 g/dl) transfusion strategy in critically ill patients. The authors noted a similar overall 30-day mortality rate between groups but a significantly lower 30-day mortality rate for less acutely ill patients in the restrictive group (Acute Physiology and Chronic Health Evaluation II score <20 and age <55 years). The mortality rate was higher in patients with significant cardiac disease treated with the liberal strategy, but the results did not achieve statistical significance (*P *= 0.69).

Mechanical ventilation is a common treatment in ICU patients with respiratory failure. A major goal of ICU care is to reduce the number of ventilator days. Numerous clinical factors have an impact on the duration of mechanical ventilation. Improving oxygen delivery to tissues is a recognized goal of ICU care, but its specific impact on outcomes in mechanically ventilated patients is not known. Anemia can lead to a reduction in oxygen delivery. The potential impact of anemia on mechanical ventilation outcomes continues to be evaluated, but there is evidence to suggest a negative impact. Nevins and Epstein [13] found that a low admission hematocrit was significantly associated with death in patients with chronic obstructive pulmonary disease receiving mechanical ventilation. Khamiees and coworkers [14] reported that mechanically ventilated patients with low hemoglobin levels are more likely to be unsuccessfully extubated than are patients with higher hemoglobin levels. Ouellette and colleagues [15] reported that a low hemoglobin level during a period of mechanical ventilation was the most significant risk factor for failure to wean from mechanical ventilation.

We hypothesized that critically ill patients requiring mechanical ventilation have an inadequate EPO response to anemia, which contributes to the development and persistence of anemia of critical illness.

## Materials and methods

The study was approved by the Institutional Review Board at Brooke Army Medical Center and was performed in accordance with the ethical standards laid down in the 1964 Declaration of Helsinki. All participants (or surrogates) were counseled and informed consent was obtained before entry into the study.

### Study patients

Adult patients (>18 years) admitted to the medical ICU of Brooke Army Medical Center with acute respiratory failure requiring mechanical ventilation for 72 hours and with a hemoglobin level below 13 g/dl were screened for eligibility. Patients with a pre-existing indication for the use of rHuEPO, including anemia associated with end-stage renal disease, cancer, or cancer therapy, and those with HIV infection treated with zidovudine were excluded. Patients with acute or chronic bleeding of any etiology and those who received rHuEPO either before admission or during the ICU course were also excluded. Transfusion thresholds and goals and mechanical ventilation management was at the discretion of the attending physician. Transfusion guidelines outlined by Hebert and coworkers [12] and the American College of Chest Physicians weaning guidelines [16] were provided as a reference, and adherence to these practices was encouraged. In total, 20 study patients were enrolled from January 2003 to December 2003.

Demographic and clinical data including Acute Physiology and Chronic Health Evaluation II scores were recorded at study entry. Admission complete blood count and basic metabolic panel were reviewed. After study enrollment, hemoglobin and EPO levels at day 3 were recorded for statistical analysis, and the arterial oxygen tension (PaO_2_)/fractional inspired oxygen (FiO_2_) ratio at day 3 was calculated. Patients were followed for the duration of mechanical ventilation.

### Control group

The control group consisted of 20 ambulatory patients with a new diagnosis of iron deficiency anemia (hemoglobin <13 g/dl, ferritin <100 ng/ml, iron <46 μg/dl) screened from a primary care clinic. All patients were free of acute illness, had normal renal function, and had not received rHuEPO during the preceding 30 days. Demographic data and hemoglobin and EPO levels were recorded for statistical analysis.

### Erythropoietin assay

Serum EPO levels were measured using a commercial two-site chemiluminescence immunoassay (Nichols Advantage Erythropoietin Assay; Nichols Institute Diagnostics, San Clemente, CA, USA) referenced to the World Health Organization recombinant DNA-derived human EPO 1st International Standard (WHO 87/684). Expected values were determined from data on 119 healthy adults (age range 18–69 years). The results ranged from <5.0 to 25.1 mU/ml. The 95% confidence interval was 5.0–24.6 mU/ml. Reproducibility was determined according to the National Committee for Clinical Laboratory Standards EP5-T2 tentative guidance document [17]. The limit of detection is estimated to be 1.2 mU/ml. The limit of detection was determined from 20 replicate determinations of the zero standard and is defined as the value two standard deviations above the mean of the 20 replicates. The functional sensitivity is estimated at 5.0 mU/ml. The functional sensitivity is based on the lowest concentration of EPO in serum where the interassay precision does not exceed a 20% coefficient of variation.

### Statistical analysis

Independent samples t-test was used to evaluate differences in age, hemoglobin, and EPO by group. Paired t-test was used to compare observed versus expected EPO levels by group. A linear regression on EPO as a function of hemoglobin level by group was performed. The results were expressed as mean ± standard deviation. *P *< 0.05 was considered statistically significant.

## Results

Twenty (5 male, 15 female; mean age 70 years, range 49–88 years) critically ill patients requiring mechanical ventilation for acute respiratory failure were enrolled in the study. Table 1 summarizes the study patients' characteristics. Of the 20 study patients, 18 had a PaO_2_/FiO_2 _ratio on day 3 of less than 300. Hemoglobin and EPO values were compared with those of 20 (5 male, 15 female; mean age 60 years, range 24–84 years) control patients with iron deficiency anemia.

There was no significant difference in hemoglobin level between the groups (mean hemoglobin 10.6 ± 1.5 g/dl in the study group versus 10.2 ± 1.0 g/dl in the control group; independent samples t-test, *P *= 0.381). Because there was no difference between groups with respect to hemoglobin, we compared the groups with respect to EPO level. A significantly lower EPO level was recorded in the mechanically ventilated patient group (mean EPO level 28.7 ± 30.4 mU/ml in the study group versus 60.9 ± 58.3 mU/ml in the control group; independent samples t-test, *P *= 0.035).

**Figure 1:**
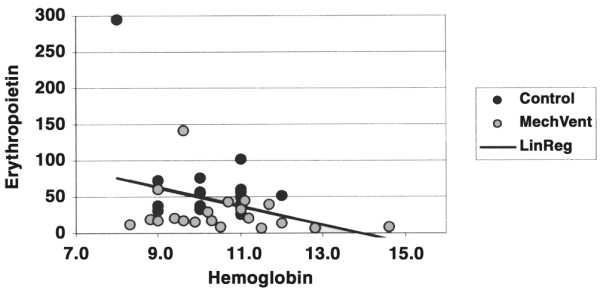
Linear regression: erythropoietin as a function of hemoglobin. The line represents the best fit to the values in the control group.

A linear regression of EPO as a function of hemoglobin was performed to confirm the difference between expected and observed EPO levels between groups (Fig. 1). There was no significant difference between the observed and expected levels of EPO in the control group (*P *= 1.000), but there was a statistically significant difference in the study group (*P *= 0.006).

## Discussion

Anemia in the ICU is a common problem, with a multifactorial etiology. We evaluated the relationship of the endogenous EPO response to anemia in the setting of mechanical ventilation and demonstrated a significantly diminished response in this population. Ambulatory iron deficient anemic patients were chosen as control patients in order to match the expected degree of anemia in ICU patients. Additionally, this population demonstrated an elevated EPO response to anemia in a previous study [3]. The EPO response in critical illness has been evaluated in specific subsets of patients but not in mechanically ventilated adult patients in a controlled design.

Rogiers and coworkers [3] compared a mixed population of critically ill patients with iron deficient control patients to determine whether a relationship between EPO response and degree of anemia existed. The study group consisted of 22 septic patients (subgroups with and without renal failure) and 14 nonseptic patients (subgroups with and without renal failure). Patients considered hypoxemic (PaO_2 _<75 mmHg) were excluded from the analysis. The control group comprised 18 ambulatory iron deficient patients without acute illness. Hematocrit values were similar between study and control patients. A significant inverse correlation between hematocrit and EPO was found in the control patients and in the nonseptic patients without renal failure. The correlation of EPO with hematocrit was lost in the septic patients and in the nonseptic patients with acute renal failure. The authors concluded that the EPO response to anemia is severely blunted in critically ill patients.

Krafte-Jacobs and coworkers [5] demonstrated a blunted EPO response in critically ill pediatric patients with acute anemia and acute hypoxia. Enrolled patients included 21 with acute anemia, 18 with acute hypoxemia (normal hemoglobin), 10 critically ill without anemia or hypoxemia, and 21 outpatients with chronic anemia but no acute illness. Hemoglobin levels were equivalent in the acutely anemic and chronically anemic patients. The EPO levels were similar in the acutely anemic, acutely hypoxemic, and critically ill control patients, but significantly less than the EPO levels in the chronically anemic patients. The authors concluded that the EPO response to known physiologic stimuli is blunted in critically ill children.

Hobisch-Hagen and coworkers [4] found no correlation between EPO and hemoglobin concentrations in 23 adult patients suffering from severe trauma. That observational study did not include a control group for comparison. Trauma patients exhibited anemia (mean hemoglobin 10.0 g/dl) on admission without significant increase during the period of observation. The mean EPO level was 49.8 U/l on day 1 without significant increase throughout the study period (to day 9). The authors concluded that patients with multiple trauma exhibit an inadequate EPO response to low hemoglobin concentrations.

In theory, the treatment of anemia in mechanically ventilated patients with respiratory failure should improve oxygen delivery to the tissues. The interplay of the other principal determinants of oxygen delivery (cardiac output and arterial oxygen saturation) and the overall impact on outcome continues to be evaluated. Hebert and coworkers [18] reported the impact of a liberal (threshold hemoglobin 10.0 g/dl, goal 10–12 g/dl) compared with a restrictive (threshold hemoglobin 7.0 g/dl, goal 7–9 g/dl) transfusion strategy in 713 mechanically ventilated patients, representing a subgroup of a larger study [12]. That study found no difference in the duration of mechanical ventilation between groups.

An adverse impact of anemia on outcome in mechanically ventilated patients has been reported. Khamiees and coworkers [14] conducted a prospective study of predictors of extubation outcome in 91 patients recovering from acute respiratory failure and who successfully completed a spontaneous breathing trial. Patients with hemoglobin values under 10 g/dl were five times as likely to have unsuccessful extubation as those patients with hemoglobin above 10 g/dl. To investigate predictors of outcome, Nevins and Epstein [13] conducted a retrospective cohort study of 166 patients with chronic obstructive pulmonary disease requiring mechanical ventilation for acute respiratory failure of diverse etiologies. Univariate analysis demonstrated lower admission hematocrit to be one of several factors associated with higher in-hospital mortality. Ouellette and colleagues [15] reported that a hemoglobin level under 9 g/dl was the most significant risk factor for unsuccessful extubation in a retrospective review of laboratory parameters and their impact on mechanical ventilation outcomes.

The etiology of anemia of critical illness remains unclear, but a blunted endogenous EPO response appears to play a role. The mechanisms that underlie the blunted endogenous EPO response also remain to be elucidated, although recent studies have demonstrated this response across a spectrum of critically ill patients, suggesting that the presence of critical illness rather than any specific diagnosis is the key factor. Patients with hypoxia – an additional stimulus for endogenous EPO production – were excluded in the aforementioned studies of adult patients. Despite the requirement for mechanical ventilation and the presence of hypoxemia (mean PaO_2_/FiO_2 _<300), the critically ill patients in our study also exhibited a blunted EPO response. These results indicate that further investigation into the etiology as well as treatment of anemia of critical ill patients should also include hypoxic patients requiring mechanical ventilation.

Limitations of our data include the small sample size and the observational nature of the study. It was not the objective of the present study to determine the clinical impact of a blunted EPO response on mechanical ventilation outcomes, which therefore cannot be addressed.

## Conclusion

In summary, we demonstrated that the EPO response to anemia in the critically ill mechanically ventilated patient is blunted, similar to findings in other previously described subsets of critically ill patients. A negative impact of anemia on outcomes in mechanically ventilated patients has been reported. Further study of therapies directed at treating anemia of critical illness and determining its potential impact on mechanical ventilation outcomes and mortality is warranted.

## Key messages

• 	Anemia in the ICU patient is a common problem with a multifactorial etiology.

• 	The EPO response to anemia in the critically ill mechanically ventilated patient is blunted.

• 	Further investigation of therapies directed at anemia of critically ill mechanically ventilated patients are necessary to determine potential morbidity and mortality benefits.

## Abbreviations

EPO = erythropoietin; FiO_2 _= fractional inspired oxygen; ICU = intensive care unit; PaO_2 _= arterial oxygen tension; rHuEPO = recombinant human erythropoietin.

## Competing interests

DRO is a member of the Speaker's Bureau and Consultant, Ortho Biotech, and is on the Speaker's Bureau, Pfizer.

## Authors' contributions

AJD modified the original protocol, executed the study, analyzed data, and drafted the manuscript. DGB assisted in executing the study, analyzing the data, and drafting the manuscript. MWQ and DEL participated in the original design and coordination of the study, and in writing the original protocol. DRO, MWQ, and DEL conceived the study. DRO assisted in the original design and drafting of the final manuscript. All authors read and approved the final manuscript.

## Figures and Tables

**Table 1:** Clinical profile of enrolled mechanically ventilated patients

Parameter	Value
*n*	20
Age (years; mean [range])	70 (49–88)
Male/female	5/15
PaO_2_/FiO_2 _ratio (mean [range])	220 (118–385)
APACHE II score (mean [range])	19.8 (8–36)
Ventilator days (mean [range])	12.3 (3–56)
Diagnosis (*n*)	
Pneumonia	8
COPD	5
Pulmonary embolus	1
CHF/cardiac ischemia	4
Acute renal failure	2

APACHE, Acute Physiology and Chronic Health Evaluation; CHF, congestive heart failure; COPD, chronic obstructive pulmonary disease; FiO_2_, fractional inspired oxygen; PaO_2_, arterial oxygen tension.
